# Microarray profiling of lung long non-coding RNAs and mRNAs in lipopolysaccharide-induced acute lung injury mouse model

**DOI:** 10.1042/BSR20181634

**Published:** 2019-04-30

**Authors:** Juan Wang, Yong-Chun Shen, Zhen-Ni Chen, Zhi-Cheng Yuan, Hao Wang, Da-Jiang Li, Kai Liu, Fu-Qiang Wen

**Affiliations:** 1 Center of Infectious Diseases, West China Hospital of Sichuan University, Chengdu 610041, China; 2 Department of Respiratory and Critical Care Medicine, West China Hospital of Sichuan University, and Division of Pulmonary Diseases, State Key Laboratory of Biotherapy of China, Chengdu 610041, China; 3 Department of Medical Affairs, West China Hospital of Sichuan University, Chengdu 610041, China

**Keywords:** Acute lung injury, Microarray analysis, Lipopolysaccharide, Long noncoding RNAs

## Abstract

Long non-coding RNAs (lncRNAs) are involved in various biological processes as well as many respiratory diseases, while the role of lncRNAs in acute lung injury (ALI) remains unclear. The present study aimed to profile the expression of lung lncRNAs and mRNAs in lipopolysaccharide (LPS)-induced ALI mouse model. C57BL/6 mice were exposed to LPS or phosphate-buffered saline for 24 h, and lncRNAs and mRNAs were profiled by Arraystar mouse LncRNA Array V3.0. Bioinformatics analysis gene ontology including (GO) and pathway analysis and cell study *in vitro* was used to investigate potential mechanisms. Based on the microarray results, 2632 lncRNAs and 2352 mRNAs were differentially expressed between ALI and control mice. The microarray results were confirmed by the quantitative real-time PCR (qRT-PCR) results of ten randomized selected lncRNAs. GO analysis showed that the altered mRNAs were mainly related to the processes of immune system, immune response and defense response. Pathway analysis suggests that tumor necrosis factor (TNF) signaling pathway, NOD-like receptor pathway, and cytokine–cytokine receptor interaction may be involved in ALI. LncRNA-mRNA co-expression network analysis indicated that one individual lncRNA may interact with several mRNAs, and one individual mRNA may also interact with several lncRNAs. Small interfering RNA (siRNA) for ENSMUST00000170214.1, - ENSMUST00000016031.13 significantly inhibited LPS-induced TNF-α and interleukin (IL)-1β production in murine RAW264.7 macrophages. Our results found significant changes of lncRNAs and mRNAs in the lungs of LPS-induced ALI mouse model, and intervention targeting lncRNAs may attenuate LPS-induced inflammation, which may help to elucidate the role of lncRNAs in the pathogenesis and treatment of ALI.

## Introduction

Acute lung injury (ALI) and acute respiratory distress syndrome (ARDS) are a sequence of lung injuries arising from a wide variety of stimulus, followed by uncontrolled inflammation, which frequently result in multiple organ dysfunction with high mortality [1]. It was estimated that the incidence of ALI is approximately 200000 with an overall mortality rate of 40% in the U.S.A. from an epidemiologic study [2]. Although significant progress has been achieved on the treatment of ALI/ARDS with mechanical ventilation or other drugs to inhibit the excessive inflammation [3,4], the prognosis of ALI/ARDS is still not optimistic. Evidence from a recent systematic review suggests that even after 2010, the overall mortality rates of ARDS in-hospital, Intensive Care Unit, 28/30 days, and 60 days were 45, 38, 30, and 32%, respectively [5]. Since the pathogenesis of ALI has not been fully elucidated, finding a novel therapeutic target for ALI/ARDS is imperatively needed.

Long non-coding RNAs (lncRNAs) are a new class of non-coding RNAs that play a role in regulating gene transcription, protein expression and epigenetic regulation at various levels. They play important roles in various diseases, including cancers, rheumatic diseases, cardiac and infectious diseases [6–10]. Our previous study observed significant changes of lncRNAs expression profiles in cigarette-smoke exposed mouse lung and revealed a potential role of lncRNAs in the pathogenesis of cigarette smoke-associated airway inflammatory disorders [11]. Huang et al. [12] also reported that the expression profiles of lncRNA were changed in the blood of pneumonia patients, suggesting that lncRNAs and their target genes may be closely associated with the progression of pneumonia. Dysregulated lncRNAs were also observed in the peripheral blood of patients with eosinophilic asthma, suggesting that lncRNAs may take part in the immune regulation of eosinophilic asthma [13]. All these studies reveal a potential role of lncRNAs in respiratory disorders. However, the potential role of lncRNAs in ALI has not been fully reported, the present study aimed to profile both lncRNAs and mRNAs in the lung of ALI mice, and tried to discuss their roles in ALI through bioinformatics analysis and cell study *in vitro*.

## Materials and methods

### Animal preparation

The study protocol was reviewed and approved by the hospital Animal Ethics Committee (2017095A).The C57BL/6 mice were prepared based on the Animal Research: Reporting of In Vivo Experiment guidelines. Ten specific pathogen-free male C57BL/6 mice (8–10 weeks, 24–26 g) were purchased and were randomly divided into two groups: control group and lipopolysaccharide (LPS)-stimulated group, each group contained five mice (Dashuo Biological Technology Co, Ltd, Chengdu, China).

### ALI model preparation

After anesthetizing with Sevoflurane Inhalation Anesthetic (Hengrui Medicine, Jiangsu, China), two group male C57BL/6 mice were challenged with phosphate-buffered saline (Life Technologies, Grand Island, NY, U.S.A.) or LPS (*Escherichia coli* O111:B4, Sigma–Aldrich, St Louis, MO, U.S.A.) through intratracheal spray using a MicroSprayer™ (PennCentury, Philadelphia, PA) [14], respectively. After 24 h, the mice were killed through intraperitoneal injection of sodium pentobarbital (100 mg/kg), followed by exsanguination from the abdominal aorta to collect lung tissue sample.

### Mouse histology

The left lung was fixed with 4% formaldehyde, and embedded by paraffin, then, Hematoxylin and Eosin (HE) stains on slices of lung tissue were used to observe the pathological changes of lung (Sigma–Aldrich, St. Louis, MO, U.S.A.), and lung injury score was assessed following the official standard of American Thoracic Society [15].

### RNA isolation

The total RNA from mouse lung tissues was extracted and purified using TRIzol reagent (Invitrogen, Carlsbad). The quantitation and quality of RNA and RNA integrity was evaluated by standard method as previously described [11].

### Microarray analysis

First, mRNA was purified from 1 mg of total RNA, each sample was amplified and transcribed into fluorescent cRNAs utilizing random primers (Arraystar Flash RNA Labeling Kit, Arraystar). Then, the cRNAs were hybridized on to the mouse LncRNA Microarray 3.0 (Arraystar). The arrays were scanned (Agilent Scanner, G2505C), and array images were analyzed (Agilent Feature Extraction Software, version 11.0.1.1). Data normalization and subsequent processing were carried out with the GeneSpring GX v12.1 software package (Agilent Technologies, Santa Clara, CA, U.S.A.).

A volcano plot filtering was used to identify differentially expressed lncRNAs and mRNAs, with the threshold defined as fold-change > 2.0 (Student’s *t*test *P*<0.05). Distinguishable lncRNA expression profile between case and control mice was showed by Hierarchical clustering. All the microarray hybridization and analysis was performed by KangChen Biotech (Shanghai, China).

### Validation of microarray results

To validate the reliability of microarray results, five up-regulated and five down-regulated lncRNAs were randomly selected to be examined by further quantitative real-time PCR (qRT-PCR), primers of the ten selected lncRNAs were listed in Supplementary Table S1.

Briefly, RNA was extracted and cDNA was synthesized using the iScript cDNA Synthesis Kit (Bio-Rad, Hercules, CA, U.S.A.). qRT-PCR was carried out by the CFX96 real-time PCR detection system using SsoFast EvaGreen Supermix (Bio-Rad, Hercules, CA, U.S.A.). Each lncRNA was quantitated by standard curve and all data were normalized to GAPDH gene expression. Student’s *t* test was used to examine the differences of lncRNA expression between ALI and controls mice (SPSS Inc., Chicago, IL, U.S.A., version of 22.0). Natural logarithm was used to analyze the relationship between fold changes of qRT-PCR and microarray analysis, a two-sided *P*<0.05 was set as significant.

### Bioinformatics analysis

Gene Ontology (GO) analysis was applied to calculate the functions of differentially expressed genes, including biological processes, molecular functions and cellular components. Signal pathway analysis was used to map genes to Kyoto Encyclopedia of Genes and Genomes (KEGG) pathways. Fisher’s exact tests were used for the statistical analyses.

### LncRNA–mRNA co-expression network analysis

The correlation between differentially expressed mRNAs and lncRNAs was evaluated by coding-non-coding gene co-expression network (CNC network) analysis. The lncRNA–mRNA pairs were identified by Pearson’s correlation coefficients (PCC) of no less than 0.9 [11]. The figure of lncRNA–mRNA co-expression network was generated by Cytoscape software (The Cytoscape Consortium, San Diego, CA, U.S.A.).

### *Cis*- and *trans*-regulated gene analysis

*Cis*-acting lncRNAs regulate the expression of genes that are positioned in the vicinity of their transcription sites, whereas *trans*-acting lncRNAs modulate the expression of genes being at independent loci [16]. The gene locations for different lncRNAs on the chromosome were determined, then the co-expressed genes (r > 0.85 or r < −0.85, *P*<0.01) located within the 300 kbp windows upstream and downstream of the differentially expressed lncRNAs were identified as the potential ‘*cis*-regulated mRNAs’. ‘trans-regulated mRNAs’ were potentially coding genes of *trans*-regulated protein as co-expressed with dysregulated lncRNAs and beyond 100 kb in genomic distance from them. To analyze functions of the potential ‘*cis*-regulated mRNAs’ or ‘*trans*-regulated mRNAs’, GO enrichment and pathway analysis was also performed.

### Cell culture and transfection

Murine RAW264.7 macrophages were purchased from Geneseed Biotechnology Co., Ltd (Guangzhou, China). Murine RAW264.7 macrophages were cultured in high-glucose DMEM (Gibco, Invitrogen Life Technologies Corporation, NY, U.S.A.) supplemented with 10% fetal bovine serum (ExCell Bio, Shanghai, China) in a humidified atmosphere with 5% CO_2_ at 37°C. Then, cells were plated in a six-well plate for further experiments.

### Cells transfection with small interfering RNA

The small interfering RNA (siRNA) duplexes for ENSMUST00000170214.1 (sequence: GCAGCAGAAGTCACTTATA) and ENSMUST00000016031.13 (sequence: AAAGAACAGGGAGCTTCAA) were commercially synthesized by Invitrogen (Cat.No. 11668019). According to the manufacturer’s instructions, RAW264.7 cells were transfected at 70% confluence with siRNA-ENSMUST00000170214.1, - ENSMUST00000016031.13 and siRNA-scrambled by Lipofectamine 2000. Twenty-four hours after transfection, RAW264.7 cells were exposed to LPS (100 ng/ml) (Sigma Chemical Co, St. Louis, MO, U.S.A.) for 24 h, and then the culture supernatants were collected for further measurement.

### Enzyme-linked immunosorbent assay measurement

The concentrations of tumor necrosis factor (TNF)-α and interleukin (IL)-1β in the culture supernatants were detected by enzyme-linked immunosorbent assay (ELISA) kit (R&D Systems, Minnesota, MN, U.S.A.) following the manufacturer’s instructions.

### Statistical analysis

All data were presented as mean ± S.D., independent *t* test was used to determine the difference between two groups. And *P*<0.05 was considered to be statistically significant. Statistical analyses were carried out with SPSS software, version 18.0 (SPSS, Inc., Chicago, IL, U.S.A.).

## Results

### Establishment of ALI mouse model

**Figure 1:**
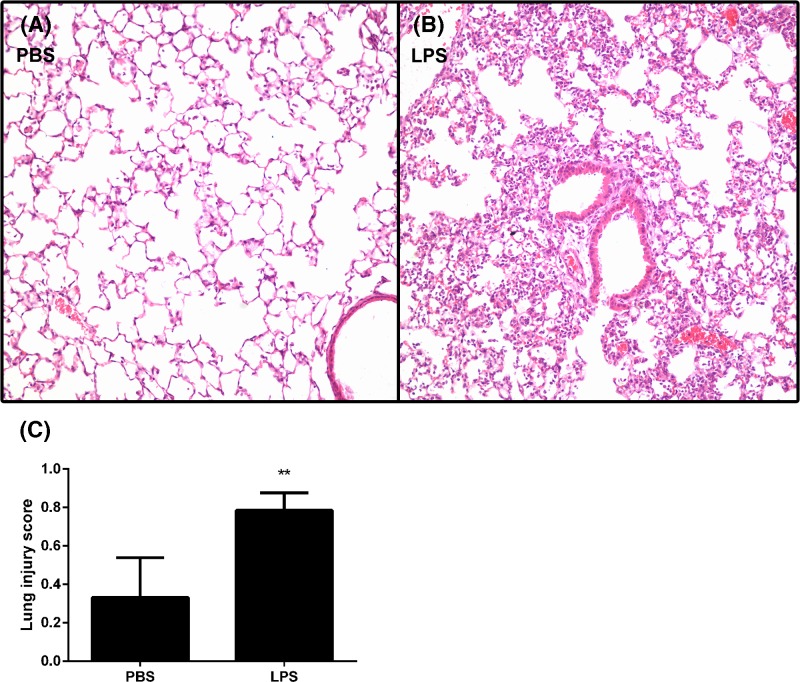
Mouse histologic changes The histologic changes were performed with HE-staining in LPS-treated mice (**A**), PBS-treated mice (**B**) and lung injury score was evaluated based on these slices (**C**). **P<0.05.

After LPS stimulation, significant inflammation was observed in the lung of ALI mouse. HE-staining showed that LPS dramatically increased the leukocyte-infiltration in mouse lungs (Figure 1A,B), ALI score increased significantly in LPS-challenged mice (*P*<0.05) (Figure 1C), suggesting successful establishment of ALI mouse model.

### LncRNA and expression profile and validation

**Figure 2:**
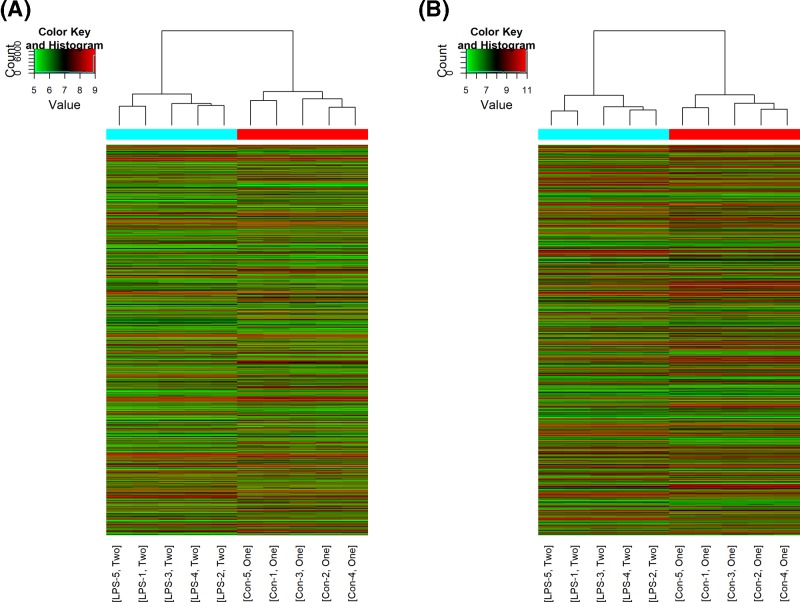
Heat maps showing the distinct lncRNA (A) and mRNA (B) expression profiles between LPS-stimulated mice and control mice Hierarchical clustering of significantly (*P*<0.05, >2-fold change) regulated lncRNAs (**A**) and mRNAs (**B**) are shown as heat maps. Expression values are presented with different colors ranging from green to red, indicating low relative expression to high relative expression, respectively (*n*=5 for control group and LPS-stimulated group, respectively).

A total of 2632 differentially expressed lncRNAs were identified in the lung tissue of LPS-exposed mice, with 1214 of them up-regulated and the rest 1418 down-regulated (fold-change > 2.0; *P*<0.05, Figure 2A). LncRNAs uc007pnu.1 (fold-change: 75.22, *P*=1.46E-07) and ENSMUST00000144634 (fold-change: 41.80, *P*=7.33E-07) were the most up- and down-regulated lncRNAs, respectively. Table 1 listed the top 20 differentially expressed lncRNAs identified by microarray analysis.

**Table 1:** The detailed information of the top ten up-regulated and top ten down-regulated lncRNAs

Probe name	Regulation	Seqname	Gene Symbol	RNA length	chrom	Fold change	*P*-value
ASMM10P051398	Up	uc007pnu.1	AK045681	2769	chr13	75.2210375	1.46509E-07
ASMM10P010513	Up	uc007rpk.1	AK145614	1723	chr13	48.3250172	8.4448E-09
ASMM10P014251	Up	AK149396	AK149396	3559	chr16	44.5581719	1.9159E-09
ASMM10P020427	Up	ENSMUST00000147219	Lcn2	1374	chr2	37.7155939	3.4948E-06
ASMM10P052114	Up	uc007rlu.1	AK089519	1193	chr13	32.6411577	7.77233E-07
ASMM10P021290	Up	uc007coi.2	Lemd1	5292	chr1	26.5422785	7.67235E-06
ASMM10P052634	Up	NR_045616	1700011B04Rik	648	chr13	24.7399888	7.0118E-08
ASMM10P051617	Up	uc007qai.1	AK161362	3212	chr13	23.6750817	2.4655E-08
ASMM10P037088	Up	AK148588	AK148588	933	chr7	18.8202011	3.6855E-09
ASMM10P002061	Up	ENSMUST00000138796	BC100530	510	chr16	18.8158229	1.10227E-05
ASMM10P020347	Down	ENSMUST00000144634	Gm13373	655	chr2	41.8033669	7.32501E-07
ASMM10P035100	Down	AK047865	AK047865	3562	chr10	30.2058144	1.00927E-06
ASMM10P035091	Down	AK081905	AK081905	2277	chr10	28.5792577	2.242E-10
ASMM10UP345	Down	uc.428+	uc.428	239	chr18	27.4789604	1.16684E-08
ASMM10P035102	Down	AK048117	AK048117	3421	chr10	26.2808024	1.99338E-07
ASMM10P035838	Down	AK132971	AK132971	2765	chr10	20.667653	1.31455E-06
ASMM10P013477	Down	ENSMUST00000159177	Fer1l6	2776	chr15	20.1880906	0.000124486
ASMM10P011787	Down	uc007tcs.1	AK047145	690	chr14	18.4579932	2.32328E-08
ASMM10P011784	Down	AK006017	AK006017	653	chr14	17.9018036	2.76482E-08
ASMM10P036217	Down	uc012fmg.1	A230057D06Rik	1713	chr7	15.5120843	2.12844E-06

These are top ten up-regulated and top ten down-regulated lncRNAs between LPS-exposed mice and controls.

### Validation of lncRNAs microarray results

**Figure 3:**
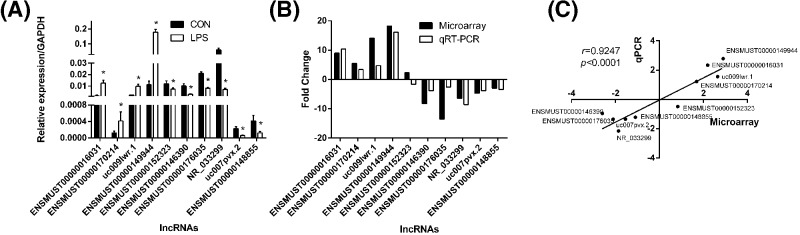
Comparison between microarray data and qRT-PCR results qRT-PCR was performed to test the differentially expressed lncRNAs between controls and LPS-stimulated mice (**A**); the fold change of each lnRNA between LPS-stimulated mice and controls was tested with microarray and qRT-PCR, respectively (**B**). The correlation between microarray and qRT-PCR was performed with natural logarithms of these different fold changes (**C**). *: *P*<0.05, *r*: standard correlation coefficient (*n*=5 for control group and LPS-stimulated group, respectively). Abbreviations: CON, control group; LPS, lipopolysaccharide group.

The expression of ten selected lncRNAs was validated by qRT-PCR (Figure 3A), and the results of microarray and qRT-PCR were compared in Figure 3B. Correlation analysis showed that the results of microarray were positively correlated with qRT-PCR (r = 0.9274, *P*<0.001, Figure 3C), suggesting the reliability of the microarray data.

### mRNA expression profile

A total of 1108 up-regulated and 1244 down-regulated mRNAs were identified in the lungs of LPS-exposed mice (fold-change > 2.0; *P*<0.05) (Figure 2B). NM_016960 (fold change: 554.10, *P*=9.12E-11) and NM_001101488 (fold change: 207.59, *P*=5.13E-11) were the most up- and down-regulated mRNAs, respectively. Table 2 summarized the top 20 differentially expressed mRNAs identified by microarray analysis.

**Table 2:** The detailed information of the top ten up-regulated and top ten down-regulated mRNAs

Probe name	Regulation	Seqname	Gene Symbol	RNA length	chrom	Fold change	*P*-value
ASMM10P005163	Up	NM_016960	Ccl20	852	chr1	554.0964063	9.12E-11
ASMM10P005162	Up	NM_001159738	Ccl20	849	chr1	135.0928322	3.4132E-09
ASMM10P028947	Up	NM_011016	Orm2	774	chr4	102.9714344	1.09916E-06
ASMM10P013206	Up	NM_030720	Gpr84	1611	chr15	94.7282259	4.85E-11
ASMM10P011036	Up	NM_053113	Ear11	722	chr14	89.7366424	9E-13
ASMM10P028944	Up	NM_008768	Orm1	768	chr4	87.3439735	7.2301E-08
ASMM10P030638	Up	NM_008599	Cxcl9	2905	chr5	80.7283366	1.52587E-06
ASMM10P006833	Up	NM_009140	Cxcl2	1083	chr5	74.3332726	2.07691E-07
ASMM10P043399	Up	NM_008694	Ngp	1176	chr9	66.9952274	6.28086E-06
ASMM10P014372	Up	NM_025288	Stfa3	412	chr16	61.2734967	9.99142E-06
ASMM10P036911	Down	NM_001101488	Gsg1l	3924	chr7	207.5899744	5.13E-11
ASMM10P021970	Down	NM_001012723	Wfdc16	1129	chr2	84.7721375	7.29101E-06
ASMM10P024917	Down	NM_007529	Bcan	3267	chr3	50.958682	7.62026E-07
ASMM10P033396	Down	NM_144943	Cd207	1530	chr6	33.2654171	4.743E-10
ASMM10P024918	Down	NM_001109758	Bcan	2685	chr3	32.2566958	1.01765E-05
ASMM10P047527	Down	NM_001099774	Krtap17-1	763	chr11	31.9873817	2.1869E-09
ASMM10P054528	Down	NM_009605	Adipoq	1233	chr16	31.1071707	3.44957E-06
ASMM10P024907	Down	NM_030707	Fcrls	1991	chr3	26.480804	4.524E-10
ASMM10P034922	Down	NM_001109749	Cntn4	5262	chr6	24.5078928	3.757E-10
ASMM10P025605	Down	NM_025285	Stmn2	1904	chr3	22.3629809	2.58718E-05

There are top ten up-regulated and top ten down-regulated mRNAs between LPS-exposed mice and controls.

### GO analysis

**Figure 4:**
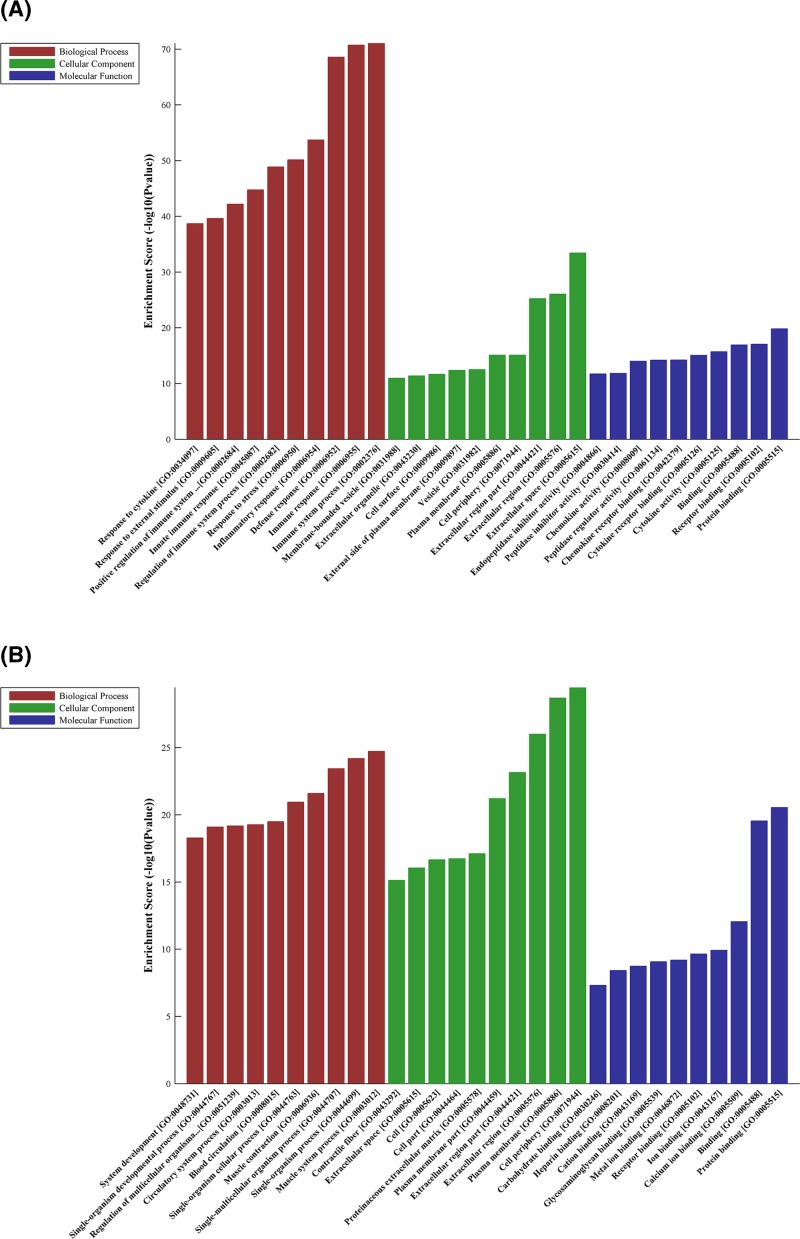
Biological functions of up-expressed and down-expressed mRNAs The most significantly up-regulated mRNAs (**A**) and down-regulated mRNAs (**B**) involved in biological process, cellular component and molecular function were identified by GO analysis.

GO analysis showed that the top three enriched biological processes of up-regulated genes were: immune system process (GO:002376), immune response (0006955), and defense response (0006952), the top three enriched cellular components of up-regulated genes were extracellular space (GO:0005615), extracellular region (GO:0005576) and extracellular region part (GO:0044421), and the top three enriched molecular function of up-regulated genes were protein binding (GO:0005515), receptor binding (GO:0005102), and binding (GO:0005488) (Figure 4A).

For down-regulated genes, the most significant enriched biological processes were muscle system process (GO:0003012), single-organism process (GO:0044699), and single-multicellular organism process (GO:0044707). The most significant enriched cellular components were cell periphery (GO: 0071944), plasma membrane (GO:0005886), and extracellular region (GO:0005576). And the most enriched molecular functions were protein binding (GO:0005515), binding (GO:0005488), and calcium ion binding (GO:0005509) (Figure 4B).

### KEGG pathway analysis

**Figure 5:**
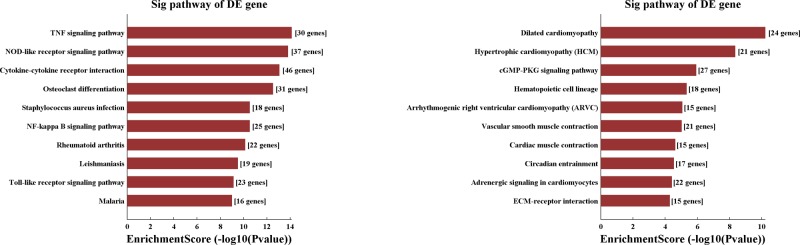
Pathway analysis for up–regulated and down–regulated mRNAs The most significant pathways related to the up-regulated genes (left) and down-regulated genes (right) were achieved by KEGG pathway analysis.

KEGG signal pathway analysis found that the main pathways of up-regulated genes are TNF signaling pathway, NOD-like receptor pathway, and cytokine–cytokine receptor interaction (Figure 5), while down-regulated transcripts in LPS-treated lung tissues are associated with dilated cardiomyopathy, hypertrophic cardiomyopathy and cGMP-PKG signaling pathway (Figure 5).

### CNC network analysis

**Figure 6:**
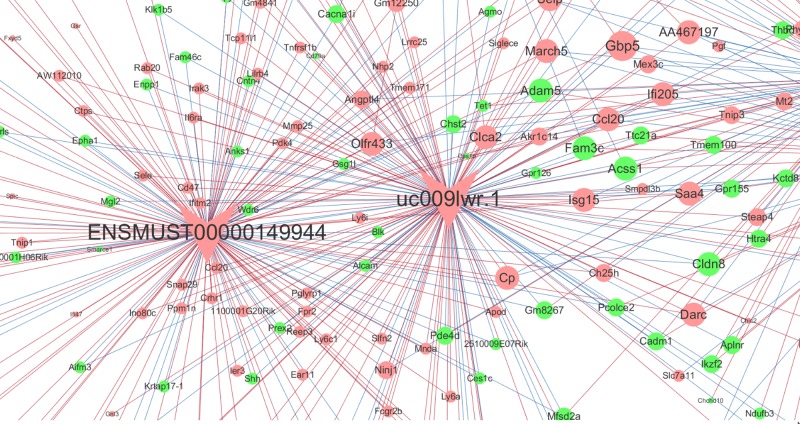
Co-expression network of the differentially expressed lncRNAs and mRNAs Round dots represent protein-coding genes and arrow represent lncRNAs. Red dots represent up-regulated genes or lncRNAs, green dots represent down-regulated genes or lncRNAs, and blue dots represent those eight tested lncRNAs. Red line represents positive correlation, and blue line represents negative correlation. The dots’ size represents the connectivity, larger dots means more genes or lncRNAs are co-expressed with this gene or lncRNA.

CNC network analysis showed all the differentially expressed lncRNAs which has a PCC value more than 0.9 with their related mRNAs. Those ten lncRNAs validated by qRT-PCR were marked as red and green. Part of results was presented in Figure 6, and the full CNC network analysis was presented in Supplementary Figure S1. Many lncRNAs were co-expressed with multiple mRNAs and lncRNAs.

### The *cis*- and *trans*-regulated genes analysis

**Figure 7:**
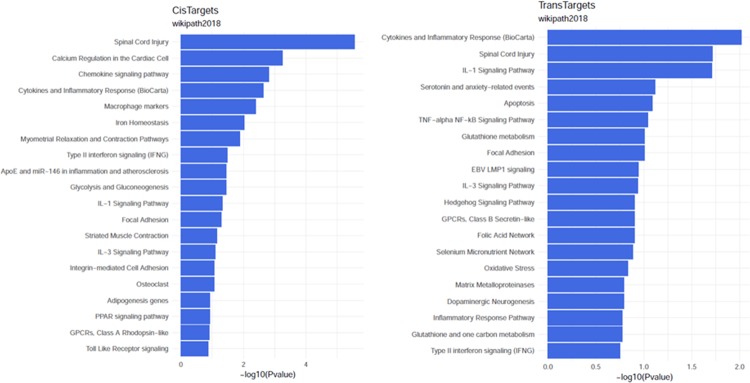
Pathway analysis of *cis*- and *trans*-regulated genes (**A**) Pathway analysis of *cis*-regulated gene. (**B**) Pathway analysis of *trans*-regulated gene.

The GO analysis of *cis*- and *trans*-regulated genes was summarized in Table 3. The pathway analysis showed that cytokines and inflammatory response, spinal cord injury, IL-1 signaling pathway, focal adhesion and IL-3 signaling pathway etc were the joint pathways for *cis*- and *trans*-regulated genes (Figure 7).

**Table 3:** The top three GO function terms for *cis-* and *trans*-regulated gene analysis

Genes	GO terms	Contents
*cis*-regulated genes		
	Biological processes	chemokine-mediated signaling pathway; positive regulation of leukocyte chemotaxis; inflammatory response
	Cellular components	specific granule; tertiary granule; tertiary granule membrane
	Molecular function	cytokine activity; chemokine activity; chemokine receptor binding
*trans*-regulated genes		
	Biological processes	response to molecule of bacterial origin; inflammatory response; calcium-mediated signaling
	Cellular components	specific granule; specific granule membrane; tertiary granule membrane
	Molecular function	cytokine activity; chemokine activity; chemokine receptor binding

### TNF-α and IL-1β measurement

**Figure 8:**
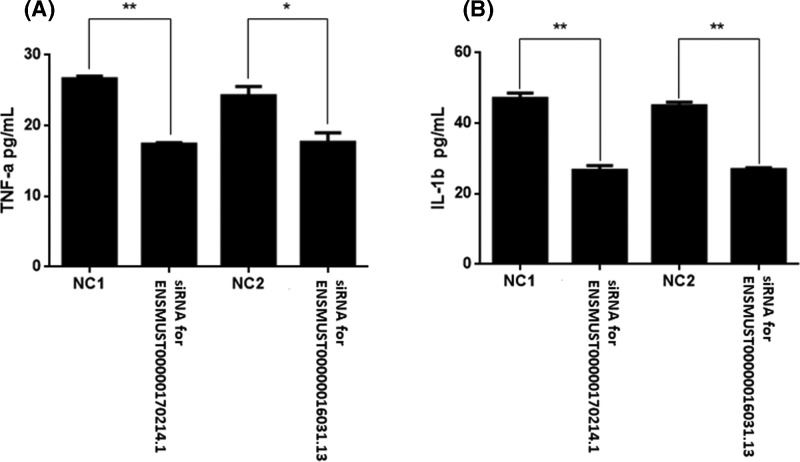
Effect of siRNAs for lncRNAs on LPS-induced **cytokines production in** murine RAW264.7 macrophages (**A**) siRNA for ENSMUST00000170214.1 significantly decreased the concentrations of TNF-α in the culture supernatants of murine RAW264.7 macrophages after stimulation of LPS for 24 h. (**B**) siRNA for ENSMUST00000016031.13 significantly decreased the concentrations of IL-1β in the culture supernatants of murine RAW264.7 macrophages after stimulation of LPS for 24 h. *P<0.05, **P<0.01.

In the present study, we chose ENSMUST00000170214.1 and ENSMUST00000016031.13 for further functional research. When compared with control group, siRNA for ENSMUST00000170214.1 or ENSMUST00000016031.13 significantly decreased the concentrations of TNF-α and IL-1β in the culture supernatants of murine RAW264.7 macrophages after stimulation of LPS for 24 h (Figure 8).

## Discussion

Recent studies on transcriptome microarray or sequencing have characterized the unique function of thousands of differentially expressed lncRNAs in the development and progression of various disorders [6–10]. However, studies regarding the role of lncRNAs in ALI are still limited. In the current study, we investigated the lncRNA expression profiles in the lung tissue of LPS-challenged mice, 2632 differentially expressed lncRNAs and 2352 mRNAs were identified via microarrays. The microarray results of randomly selected lncRNAs were further validated by qRT-PCR analysis, and the data from qRT-PCR analysis matched well with those from microarray assay. In addition, study *in vitro* suggests that lncRNAss may play a role in the treatment of ALI through the regulation of inflammation. Our study indicated that there may be a potential role of lncRNAs in the pathogenesis and therapy of ALI.

Previous studies have confirmed that lncRNAs participate in various physiologic or pathologic processes at different levels, including chromatin remodeling, regulation of gene transcription, protein expression and epigenetic regulation [17]. Accordingly, GO analyses were performed to determine the potential roles of the differentially expressed genes. Our data revealed that the up-regulated mRNAs are mainly involved in immune system process, immune response and defense response. It has been widely accepted that immune system and host defense were involved in the onset of ALI/ARDS, the immune system contains diverse cell types that coordinate responses to infection [18]. LncRNAs may play key roles in epigenetic and transcriptional regulation, and have shown great potential as key regulatory molecules of immune cell gene expression programs in response to microbial-derived clues [19]. Previous studies showed that lncRNAs regulate the LPS-stimulated inflammatory response in human monocytes, and lncRNAs would be important regulators of human innate immune response [20]. Our data strongly supported these previous studies, indicating lncRNAs-mediated immune defense mechanism participates in the LPS-induced ALI model and may offer clues for early intervention of ALI through lncRNAs.

KEGG analysis showed most up-regulated genes were related to TNF signaling pathway and NOD-like receptor. TNF families have been found to provoke the release of downstream inflammatory cytokines, thus further mediating the innate immune response and inflammatory process in ALI [21]. Recent study reported that lncRNA-HOTAIR increase the release of TNF-α in the cardiomyocytes of LPS-induced sepsis mice by activating NF-κB through the phosphorylation of NF-κB p65 subunit, suggesting that lncRNA may plays a role in ALI through the regulation of TNF-α [22]. Growing studies revealed that both (NOD-) like receptor protein 9b and (NOD-) like receptor protein 3 play a role in the regulation of ALI [23,24], and these findings supported our bioinformatics results.

Based on current evidence, most of the lncRNAs may function locally to activate or suppress the expression of their neighboring or overlapping gene [25]. Thus, the potential *cis*- or *trans*-target genes for different expressed lncRNAs were predicted to investigate the possible significance of these lncRNAs in the lung response to LPS stimulus. And GO analysis found that cytokines, chemokines, and inflammatory response are involved in the pathogenesis of ALI, which were confirmed by previous publications [26,27]. Signal pathway analysis found that both *cis*- and *trans*-regulated genes are involved in pathways of cytokines and inflammatory response, IL-1 signaling pathway, focal adhesion, and IL-3 signaling pathway, based on previous findings, these pathways are critical for development of ALI [26,28,29], suggesting dysregulated lncRNAs may play a role in ALI/ARDS through such signal pathways enriched by *cis*- or *trans*-target genes.

Moreover, based on CNC network analysis, we found that many lncRNAs were significantly related to the expression of multiple protein-coding genes, and different lncRNAs were differentially correlated with the same genes. Notably, uc009lwr.1 was positively, while ENSMUST00000146390 and NR_033299 were negatively related to CXCL-1 and CXCL-2. These two chemokines have been found to be involved in the pathogenesis of ALI. CXCL-1 is released by the activated neutrophils, and then mediates neutrophilic airway inflammation [30]. CXCL-2 is secreted by monocytes, and trigger downstream inflammatory response [31]. Herein, our date implied that these lncRNAs might play different roles in the immune responses during ALI. Specifically, uc009lwr.1 would promote the onset of inflammatory responses, while ENSMUST00000146390 and NR_033299 would play protective role against ALI-related inflammation. These findings supported the results of GO and KEGG pathway analyses, indicating the critical role of innate immune response in LPS-induced inflammatory response. CNC analysis suggests most lncRNAs were co-expressed with multiple mRNAs and lncRNAs, indicating that multiple *trans*-regulative mechanisms were involved. Further studies should be performed to explore the underlying mechanisms of these differentially expressed lncRNAs.

To further confirm the role of lncRNAs in ALI, we performed a study in *vitro* to investigate whether interventions targeting on lncRNAs plays a role in the protection of ALI, and we used siRNAs on two PCR-verified lncRNAs, ENSMUST00000170214.1 and ENSMUST00000016031.13, and we observed that the interfering of ENSMUST00000170214.1 and ENSMUST00000016031.13 decreased LPS-induced TNF-α and IL-1β production, considering the important role of TNF-α and IL-1β in ALI [32,33], we proposed that therapy targeting lncRNAs may provide novel direction for the treatment of ALI/ARDS. And more studies *in vivo* and clinical research should be performed to investigate therapeutic potential of lncRNAs in ALI/ARDS.

Taken together, thousands of lncRNAs and mRNAs in the lung were differentially expressed after LPS treatment. Bioinformatics analyses revealed that different lncRNAs would exhibit diverse potential functions which were related to differentially expressed genes. Study *in vitro* suggests the intervention on lncRNAs may attenuate inflammation in ALI/ARDS. However, further studies need to be implemented to investigate the molecular mechanisms and biological functions of lncRNAs and determine whether they can serve as novel therapeutic targets in ALI.

## Supporting information

**Supplementary Figure 1:** 

**Supplementary Table 1:** The primer sequences of validated LncRNAs in this study

## Abbreviations

ALI: acute lung injury
ARDS: acute respiratory distress syndrome
CNC network: coding-non-coding gene co-expression network
GO: gene ontology
HE: Hematoxylin and Eosin
IL: interleukin
KEGG: Kyoto Encyclopedia of Genes and Genomes
lncRNA: long non-coding RNA
LPS: lipopolysaccharide
qRT-PCR: quantitative real-time PCR
siRNA: small interfering RNA
TNF: tumor necrosis factor
