# Supplementary material for: Microarray profiling of lung long non-coding RNAs and mRNAs in lipopolysaccharide-induced acute lung injury mouse model
Source: Biosci Rep. 2019 Apr 30;39(4):BSR20181634. doi: 10.1042/BSR20181634 (PMC6488857; doi:10.1042/BSR20181634)
Supplement: Supplementary file 1 [file bsr20181634_Supp1.pdf]

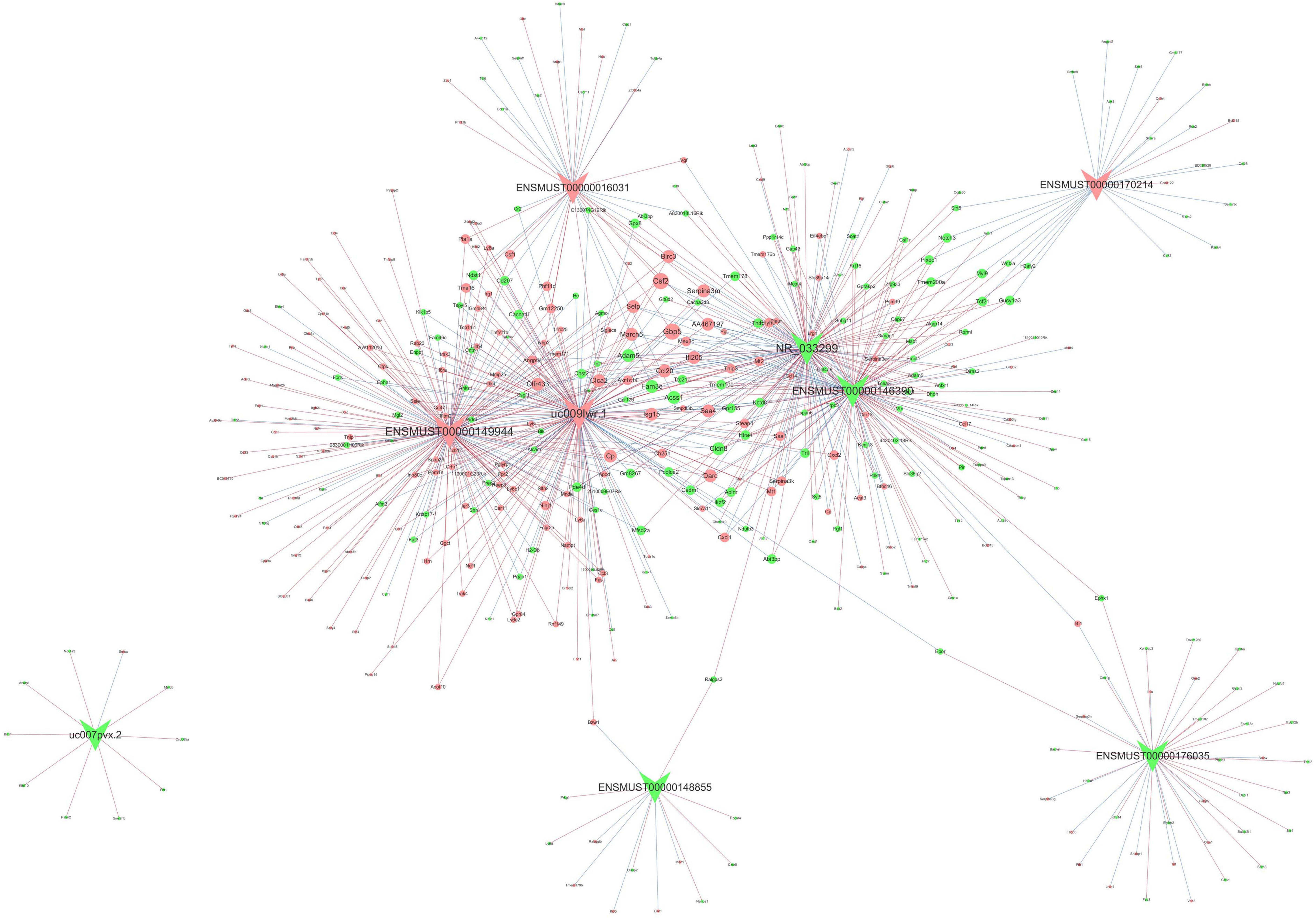

ENSMUST00000016031
ENSMUST00000170214
NR_033299
ENSMUST00000146390
uc009lwr.1
ENSMUST00000149944
uc007pvx.2
ENSMUST00000148855
ENSMUST00000176035

Supplementary Table 1. The primer sequences of validated LncRNAs in this study

LncRNA seqname	Associated_protein_name	Forward (5'-3')	Reverse (5'-3')
ENSMUST00000149944	formyl peptide receptor 2	ACAGGAACCGAAGAGTGTAAGA	CTCCAGGAATTCATTGAGAGGA
ENSMUST00000152323	angiotensin II, type I receptor-associated protein	GATTGGACCTGGTGTGCTGAGT	CCACGAATCTTCTGCCTACACT
ENSMUST00000170214	leucine zipper transcription factor-like 1	TTCTGCTTTGGTTTAGACTTCC	CATCCTTTTGCGTTAGTGAGTG
uc009lwr.1	lipoprotein lipase	GAAAACAAAATGGGACACCG	GGAAACTGATTTCTGCTCTGG
ENSMUST00000016031	histidine ammonia lyase	TGGTGATTGAAGGTGATGTGAT	TCCCTGTTCTTTCTTTGATGAT
ENSMUST00000176035	archaelysin family metallopeptidase 1	AAGGGAGACATTTGAGAAGGAA	TTCTGTCAGTGAGACTCGCTTT
ENSMUST00000146390	somatostatin receptor 2	GCGACAGTAAGCAGGACAAA	CGGACAAGCCAGACTCAAA
NR_033299	RAR-related orphan receptor alpha	TTGTGATGTAAATGGTGCCC	GGTGTTTGTGGTAGGAGGTGTA
uc007pvx.2	cytidine monophospho-N-acetylneuraminic acid hydroxylase	GGCAGAAGTCGGTCCTCTATTC	AAGTACCCAGCAAACGGACAGT
ENSMUST00000148855	kynureninase (L-kynurenine hydrolase)	ATCTTAAATTTCAGGTTAGTGGG	GAAAGACCTCTAAACTGGCG
